# The relationship between the development trajectory of symptom burden and exercise adherence during remote pulmonary rehabilitation in olderly patients with COPD

**DOI:** 10.3389/fmed.2025.1665349

**Published:** 2025-09-26

**Authors:** Li Feng, Haiyan Ji, Qing-qing Yang, Mengyao Liang

**Affiliations:** ^1^Department of Nursing, The Sixth People's Hospital of Nantong, Nantong, Jiangsu, China; ^2^Department of Respiratory Medicine, The Sixth People's Hospital of Nantong, Nantong, Jiangsu, China

**Keywords:** COPD, remote, pulmonary rehabilitation, symptom burden, exercise compliance

## Abstract

**Objective:**

To investigate the dynamic relationship and interaction between symptom burden and exercise adherence in olderly patients with Chronic obstructive pulmonary disease (COPD) undergoing remote pulmonary rehabilitation, and to establish a foundation for enhancing remote rehabilitation interventions.

**Methods:**

A total of 340 olderly patients with COPD, admitted to the Respiratory Center of Nantong Sixth People’s Hospital between May 2023 and February 2025, were selected using a convenience sampling method. The Symptom Burden Scale (SSS-8) and Exercise Adherence Scale were employed to assess symptom burden and exercise adherence levels at baseline (T1), 5 weeks (T2), 9 weeks (T3), and 12 weeks (T4). A cross-lag model was constructed to analyze the causal relationship between these variables.

**Results:**

Symptom burden exhibited a decreasing trend at each stage (*F* = 36.74, *p* < 0.001). Exercise compliance demonstrated a gradual increase (*F* = 38.92, *p* < 0.001). The cross-lag model indicated that symptom burden and exercise compliance in the early stage (T1-T2) were mutually causal (*β* = −0.32, *p* = 0.002; *β* = −0.24, *p* = 0.011). Symptom burden in the middle stage (T2-T3) significantly negatively predicted exercise compliance (*β* = −0.39, *p* < 0.001). No significant predictive relationship was found between them in the late stage (T3-T4; *p* > 0.05).

**Conclusion:**

The symptom burden of olderly patients with COPD during remote pulmonary rehabilitation is moderate to severe, while exercise compliance is at a moderate level. The dynamic relationship between symptoms and behavior shifts from a bidirectional effect in the early stage to symptom dominance in the middle stage, with no influence in the later stage. It is essential to implement precise interventions tailored to the characteristics of the three-stage trajectory, addressing both symptoms and behavioral obstacles in the early stage, focusing on symptom management in the middle stage, and reinforcing behavioral habits in the later stage.

## 1 Introduction

Chronic obstructive pulmonary disease (COPD) is a globally prevalent chronic respiratory illness characterized by persistent airflow limitation. It has a high prevalence, leads to disability, and has significant mortality rates, posing a substantial health burden, particularly for the olderly, with its prevalence increasing (1, 2). COPD is characterized by a prolonged disease course and recurrent symptoms. Patients often experience a burden of multiple symptoms, including dyspnea, shortness of breath, cough, expectoration, and fatigue, which seriously affect daily activities and quality of life (3). Pulmonary rehabilitation, a key non-pharmacological intervention in the management of COPD, has been demonstrated to effectively alleviate symptoms, enhance exercise endurance, and improve the quality of life (4). In recent years, Tele-Pulmonary Rehabilitation has developed rapidly as an innovative rehabilitation model, particularly suitable for olderly COPD patients with inconvenient travel or limited resources (5). It enables patients to receive professional rehabilitation guidance and supervision within a home setting, theoretically ensuring ongoing access to rehabilitation (6). However, this environment, which lacks face-to-face direct monitoring and immediate feedback from medical staff, imposes higher demands on patients’ abilities to perceive and manage symptoms at home, as well as their adherence to exercise programs (7).

The symptom burden is not static during pulmonary rehabilitation. Over 80% of COPD patients experience a daily symptom burden, influenced by various factors including disease fluctuation, environmental influences, comorbid conditions, and psychological status (8). The symptom burden experienced by patients during remote rehabilitation shows a dynamic and evolving development trajectory and varies among individuals (9). Exercise training constitutes the cornerstone of a pulmonary rehabilitation program, and patient compliance with the exercise regimen is essential for its effectiveness (10). Exercise adherence pertains to the congruence between the physical activities undertaken by the patient and the prescribed activities of the rehabilitation program. Maintaining good exercise adherence is particularly challenging and critical when operating in a remote mode without direct supervision (11). Current theories and research indicate that symptom burden may be a significant factor impeding patients’ adherence to exercise. For instance, severe dyspnea can lead to a fear of activity, which in turn diminishes exercise engagement (12–14). Conversely, regular and sufficient exercise may improve symptom perception. However, most current studies on the symptom burden and exercise compliance of COPD patients, both domestically and internationally, primarily concentrate on cross-sectional surveys or short-term observations (15, 16). These methods are insufficient for capturing the dynamic patterns of these changes over time during rehabilitation. The text primarily addresses static correlation, yet there is an insufficient examination of how the developmental path of symptom burden influences subsequent dynamic alterations in exercise adherence, and how the trajectory of exercise adherence, in turn, affects the subsequent path of symptom burden through feedback mechanisms. Especially within the context of emerging remote pulmonary rehabilitation, the dynamic interaction among olderly individuals has not been clearly defined. Consequently, this study aims to employ a longitudinal tracking design and a latent growth model to model and analyze the data of olderly COPD patients throughout the entire remote pulmonary rehabilitation program. The aim was to identify the trajectory patterns of symptom burden and exercise adherence over time in different patient groups. The association between symptom burden trajectory and exercise adherence trajectory was further explored, with special attention to whether the symptom burden trajectory had a predictive effect on the trajectory of exercise adherence in the subsequent period, and whether the exercise adherence trajectory had a lagged effect on the subsequent evolution of the symptom burden trajectory.

The findings of this study will elucidate the interaction mechanism between the core symptom experience and changes in exercise behavior over time in olderly patients with COPD undergoing remote pulmonary rehabilitation. This will enable clinical medical staff to issue timely warnings and dynamically adjust rehabilitation strategies in response to alterations in the patients’ symptom trajectories during remote management. Additionally, the study aims to offer a significant theoretical foundation and practical guidance for overcoming critical barriers, enhancing compliance, and ultimately improving the overall effectiveness of remote pulmonary rehabilitation.

## 2 Subjects and methods

### 2.1 Research subjects

This study was designed as a prospective observational cohort study conducted at the Respiratory Center of the Sixth People’s Hospital of Nantong between May 1, 2023, and February 20, 2025. Consecutive convenience sampling was used to recruit elderly COPD patients who were receiving stable-phase management and participating in a hospital-led structured tele-rehabilitation program. The sample size was estimated using the Monte Carlo simulation method, referencing literature on the correlation between “symptom burden” and “exercise adherence” in patients with chronic diseases. The correlation coefficients typically ranged from 0.3 to 0.6. The study’s power analysis was conducted using G*Power 3.1 software, with an expected medium effect size (f^2^ = 0.15), a significance level of *α* = 0.05, a statistical power of 1-*β* = 0.95, and a latent growth model for planned analysis, indicating a minimum required sample size of approximately 250–300 participants. Considering the complex modeling requirements for individual repeated measurement data in trajectory analysis and the potential loss to follow-up, the initial target sample size was set as 360. Inclusion criteria: ① age 60 or higher; ② patients with COPD who met the Global Initiative for Chronic Obstructive Lung Disease (GOLD) criteria and were clinically stable (defined as no acute exacerbation requiring hospitalization or emergency treatment in the past 4 weeks); ③ volunteer to participate in a standardized remote pulmonary rehabilitation (Tele-PR) program in a hospital for at least 12 weeks and have the basic conditions needed to complete the program (such as having a smartphone/tablet/computer that can run rehabilitation apps, and being able to conduct exercise training at home); ④ clear consciousness, no severe hearing or vision impairment affecting communication, able to understand the research content and complete the electronic questionnaire/APP data reporting and assessment tools independently or with the assistance of researchers or family members. Exclusion criteria: ① Patients with severe cognitive impairment (such as MMSE ≤ 23) or definite diagnosed mental diseases (such as dementia, schizophrenia, severe depression, etc.), unable to understand and follow rehabilitation guidance and complete the assessment; ② Presence of severe and uncontrolled comorbidities that significantly limit exercise capacity or constitute contraindications to exercise (e.g., unstable angina, recent myocardial infarction (within 6 months), severe heart failure (NYHA class III–IV), uncontrolled severe arrhythmias, risk of pulmonary hypertension crisis, severe cor pulmonale, active deep vein thrombosis, severe osteoporosis prone to fractures, severe limb mobility impairment (such as hemiplegia or paraplegia), or resting SpO₂ < 90% despite oxygen therapy); ③ Complicated with other serious diseases affecting respiratory function. This study was approved by the Ethics Committee of Nantong Sixth People’s Hospital (Approval number: NTLYLL2023061). All patients provided informed consent.

### 2.2 Remote pulmonary rehabilitation intervention program

The remote pulmonary rehabilitation program evaluated in this study was conducted at the Respiratory Center of the Sixth People’s Hospital of Nantong, which comprises four respiratory departments. Following an initial screening and the creation of patient files by outpatient specialists, all participants undertook a 12-week structured course (consisting of 24 to 36) sessions, structured as follows: (1) Baseline assessment and the creation of an individualized plan: Lung function (GOLD stage), 6-min walk distance (6MWD), and modified Medical Research Council (mMRC) were evaluated by independent physicians. Electronic health records were established, and risk stratification schemes were developed. (2) Structured exercise training: This included endurance training (e.g., bed bicycle, Borg scale 3–4), resistance training (using elastic bands at 40% of 1RM), and respiratory muscle training (with a threshold loading device). (3) Self-management education: This encompassed the standardized use of medication, symptom coping strategies (such as graded management of fatigue), and early identification of acute exacerbations. (4) Dynamic quality control: Nursing staff confirmed patient participation by telephone the night before the course, distributed video course packages via WeChat, and inquired about the patient’s completion status by telephone. For patients requiring on-site guidance for pulmonary rehabilitation, COPD “Internet + nursing service” could be accessed through mobile phones.

Standard Protocol for the 6-Minute Walk Test (6MWT): The 6MWT in this study was strictly conducted in accordance with the standard protocol outlined by the American Thoracic Society (ATS) guidelines. The test was performed in a 30-meter-long, quiet, flat, and hard-surfaced enclosed corridor. Prior to the test, patients were required to rest for at least 15 min. A trained researcher provided standardized instructions to all participants to ensure consistency in guidance. During the test, participants were permitted to use their routine oxygen therapy, but no encouragement was offered. A portable pulse oximeter was used to monitor and record heart rate, SpO₂, and Borg Rating of Perceived Exertion (RPE) at baseline, immediately after the test, and at the 1st and 3rd minutes of recovery. Each patient underwent two tests at baseline, separated by an interval of at least 30 min to allow sufficient rest. The longer distance achieved in the two tests was adopted as the final result to minimize the learning effect and enhance the reliability of the outcome. Studies on the intervention components have been published in domestic journals (17–20).

### 2.3 Research tools

#### 2.3.1 General information questionnaire

It includes (1) Demographic and sociological characteristics: age, gender, educational level, and who lives with; (2) Disease-related information: duration since COPD diagnosis, whether long-term home oxygen therapy is used, major comorbidities, GOLD classification, and baseline 6-min walk distance; (3) Exercise environment: whether there is exercise space at home and whether basic rehabilitation equipment is available.

#### 2.3.2 Symptom burden scale

The Somatic Symptom Scale-8 (SSS-8) was utilized to evaluate somatic symptom distress over the past 7 days (21). The scale consists of eight items, encompassing gastrointestinal issues, back pain, limb or joint pain, headaches, chest tightness or shortness of breath, dizziness, fatigue or lack of energy, and sleep difficulties. Each item is scored on a Likert scale ranging from “not at all” to “extremely troubled,” with values from 0 to 4 points, resulting in a total score between 0 and 32. Severity levels are categorized as follows: 0 to 3 (none or very low), 4 to 7 (mild), 8 to 11 (moderate), 12 to 15 (high), and 16 to 32 (severe). The scale demonstrates good reliability and validity, with a Cronbach’s alpha coefficient of 0.715 in this study.

#### 2.3.3 Exercise compliance scale

It was compiled by Weng Guizhen (22) in 2014. The scale comprised four dimensions: physical exercise compliance (8 items), exercise monitoring compliance (3 items), actively seeking advice compliance (4 items), and an additional dimension (15 items). Each item was scored using the Likert 4-point scale (ranging from 1 to 4 points), with a total possible score of 60 points. The exercise compliance rate was calculated as follows: (actual compliance score / maximum theoretical compliance score) × 100%. Compliance rates were categorized into three levels: high (≥ 75.0%), medium (≥ 50.0%), and low (< 50.0%). The Cronbach’s alpha coefficient for the scale in this study was 0.801.

### 2.4 Timing of the investigation

Studies have found (23) that pulmonary rehabilitation exercises yield better results within 6–12 weeks, and the longer the duration, the more pronounced the benefits. In this particular study, the pulmonary rehabilitation program lasted 12 weeks. To evaluate various stages of the program, follow-up time points were selected at 1–3 days before the start of tele-rehabilitation (T1), after 5 weeks of rehabilitation (T2), after 9 weeks (T3), and upon completion at 12 weeks (T4). These time points were chosen to assess baseline status, early adaptation, mid-term progress, and the final outcomes of the program. Apart from the initial general data collection, symptom burden and exercise adherence were measured at subsequent time points.

### 2.5 Data collection methods

The research data collection was completed by the research group’s members, who had undergone standardized training. This group included two respiratory nurses, one rehabilitation therapist, and two graduate students. The training encompassed a detailed explanation of the research protocol, interpretation of questionnaire items, standardized communication skills, operation guidelines for the remote pulmonary rehabilitation course, ethical norms, and emergency handling procedures. Following the training, a simulation assessment was conducted to ensure the consistency and professionalism of the data collection process. Prior to the formal launch, a small sample was recruited to test the entire process (including platform operation, scale understanding, and follow-up skills). This was done to optimize the operational path for olderly patients, simplify the questionnaire items, and confirm the feasibility of remote pulmonary rehabilitation. The general data form was independently entered by two researchers, and a third-party review was initiated when the discrepancy rate exceeded 5%. The research assistant verified the data integrity daily and implemented a three-tier intervention for patients missing more than three rehabilitation sessions: ① an APP push reminder, ② WeChat notification to family members, and ③ a telephone follow-up by researchers. The study’s prevention and management strategy for loss to follow-up was as follows: First, an emergency contact was established and a commitment to participate was signed. Second, failure to submit the core data (symptom scale + exercise compliance questionnaire) at two consecutive time points was deemed loss to follow-up. A total of 340 valid samples, who completed the entire data collection at four time points, were ultimately included in the study. The reasons for loss to follow-up were as follows: technical issues (equipment failure/inability to perform), health events (acute exacerbation/hospitalization), subjective withdrawal (unwillingness/time), and loss of contact.

### 2.6 Statistical methods

A stratified data analysis strategy was employed in this study. All data were verified by two individuals and inputted into Excel 2021 to create a database. Basic statistical analysis was conducted using SPSS 26.0. Categorical data were characterized by frequency (n) and percentage (%). Continuous variables with a normal distribution were presented as mean ± standard deviation (x̄ ± s). Repeated Measures ANOVA was utilized to assess the dynamic change trends of symptom burden and exercise compliance at T1 (baseline), T2 (5 weeks), T3 (9 weeks), and T4 (12 weeks), with the Greenhouse–Geisser correction applied when the sphericity assumption was violated. To further investigate the dynamic interaction between symptom burden and exercise compliance, Mplus 8.7 was utilized to construct a dual-model system. A Cross-Lagged Panel Model (CLPM) was implemented to examine the bidirectional predictive relationship between symptom burden and exercise adherence, while controlling for the effects of age, GOLD stage, and baseline 6-min walk distance. The Parallel Process Latent Growth Model (PPLGM) was used to define the latent trajectories of symptom burden and exercise adherence, including intercept and slope factors, to quantify the association between initial levels of symptom burden and exercise adherence, the covariation of change rates, and the prediction effect across trajectories. Strict criteria were applied for model fit: χ^2^/df < 3, Comparative Fit Index (CFI) > 0.95, Tucker-Lewis Index (TLI) > 0.95, Root Mean Square Error of Approximation (RMSEA) < 0.06 (with the upper limit of the 90% confidence interval <0.08), and Standardized Root Mean Square Residual (SRMR) < 0.08. Missing data were handled using the full information maximum likelihood method (FIML). All statistical tests were two-tailed, with the significance level set at *α* = 0.05.

## 3 Results

### 3.1 General information of patients

A total of 340 olderly patients with COPD who completed the baseline assessment were enrolled in this study to participate in remote pulmonary rehabilitation. The average age of the patients was 72.4 ± 6.8 years, with males comprising 63.5% of the participants. The patients had a mean duration of 8.3 ± 4.6 years since diagnosis; details are provided in Table 1.

**Table 1:** General baseline characteristics of olderly COPD patients undergoing remote pulmonary rehabilitation (*N* = 340).

Feature category	Number	Percentage (%)
Gender		
Male	216	63.5
Female	124	36.5
Degree of education		
Primary school and below	109	32.1
Junior high school	94	27.6
High school or technical secondary school	89	26.2
College or above	48	14.1
With whom to live		
Live alone	32	9.4
Spouse and children	301	88.5
Other	7	2.1
Duration of COPD diagnosis (years)		
<5	102	30.0
5–10	148	43.5
>10	90	26.5
Long-term home oxygen therapy		
Yes	75	22.1
No	265	77.9
Main complications (multiple choices)		
Hypertension	143	42.1
Coronary heart disease	81	23.8
Diabetes	67	19.7
Osteoporosis	49	14.4
Other	92	27.1
GOLD classification		
Class I	62	18.2
Class II	177	52.1
Class III	101	29.7
Baseline 6MWD (x̄ ± s)	326.4 ± 85.2	
6MWD group		
< 350 m	148	43.5
350–450 m	153	45.0
>450 m	39	11.5
Home exercise space		
There are fixed areas	215	63.2
No fixed area	125	36.8
Equipped with basic rehabilitation facilities		
Elastic band	198	58.2
Dumbbell	121	35.6
No equipment	89	26.2

### 3.2 Trends in symptom burden and exercise compliance at four time points

**Figure 1:**
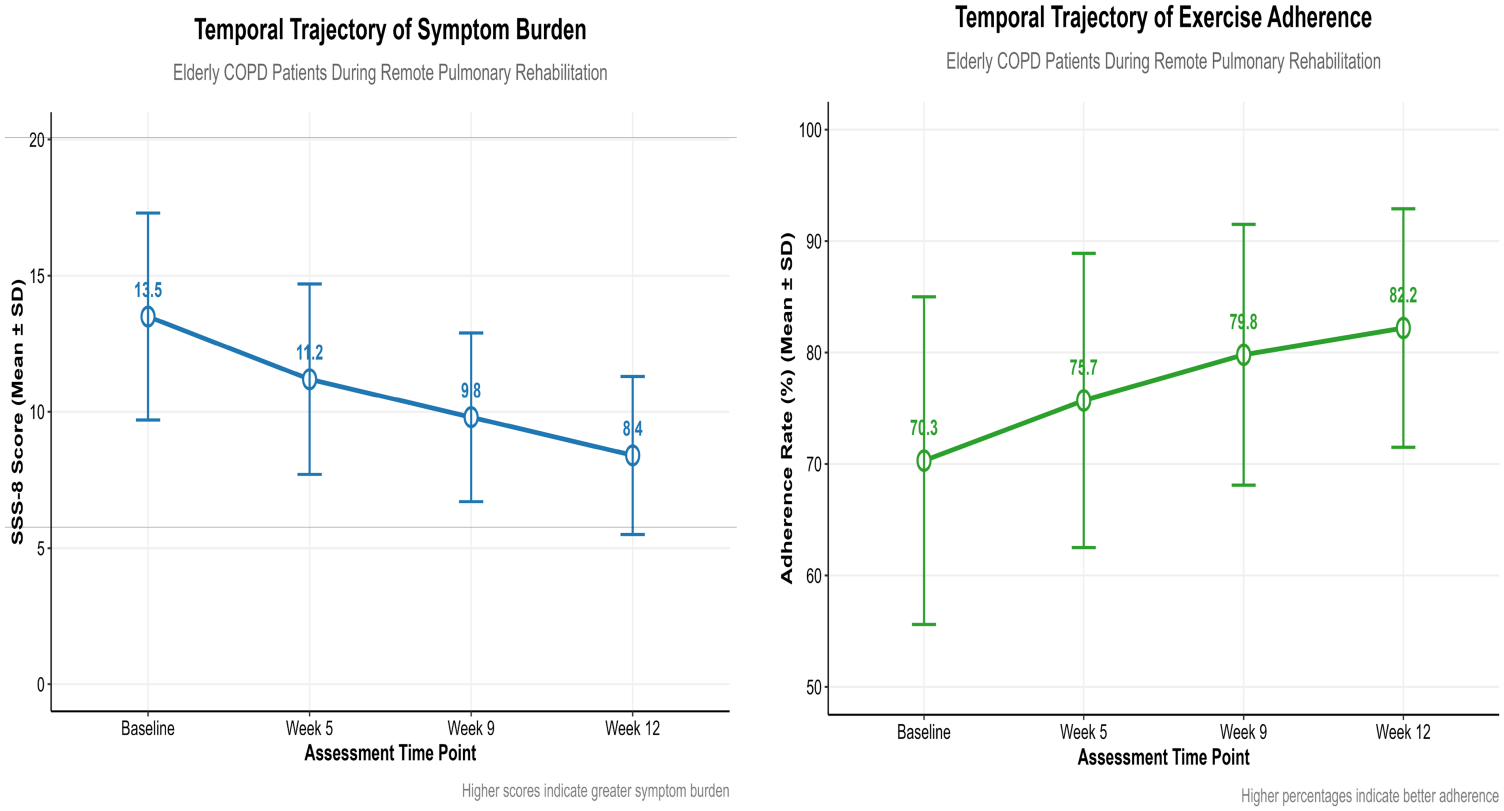
Trend chart of symptom burden and exercise adherence.

Overall, the SSS-8 total score decreased significantly from 13.5 ± 3.8 at baseline to 8.4 ± 2.9 at 12 weeks (*F* = 36.74, *p* < 0.001), and the severity also changed (χ^2^ = 118.6, *p* < 0.001). The score of exercise compliance increased from 42.2 ± 8.8 to 49.3 ± 6.4 (*F* = 42.15, *p* < 0.001), the compliance level was significantly optimized (χ^2^ = 156.3, *p* < 0.001), and the high-compliance group expanded from 30.9 to 72.9%, showing an overall upward trend. See Table 2 and Figure 1 for details.

**Table 2:** Dynamic changes in symptom burden and exercise adherence during remote pulmonary rehabilitation in olderly COPD patients (*N* = 340).

Variable	T1	T2	T3	T4	F/χ^2^ value	*p*
1. SSS-8						
Total points (x̄ ± s)	13.5 ± 3.8	11.2 ± 3.5	9.8 ± 3.1	8.4 ± 2.9	F = 36.74	< 0.001
Classification proportion [n(%)]					χ^2^ = 118.6	< 0.001
Low (0–3)	2 (0.6)	5 (1.5)	12 (3.5)	23 (6.8)		
Mild (4–7)	8 (11.2)	72 (21.2)	109 (32.1)	142 (41.8)		
Moderate (8–11)	148 (43.5)	167 (49.1)	155 (45.6)	135 (39.7)		
Height (12–15)	105 (30.9)	79 (23.2)	56 (16.5)	35 (10.3)		
Severe (16–32)	47 (13.8)	17 (5.0)	8 (2.4)	5 (1.5)		
2. Exercise compliance						
Total score (x̄ ± s)	42.2 ± 8.8	45.4 ± 7.9	47.9 ± 7.0	49.3 ± 6.4	F = 42.15	< 0.001
Compliance Rate (%)	70.3 ± 14.7	75.7 ± 13.2	79.8 ± 11.7	82.2 ± 10.7	F = 38.92	< 0.001
Proportion of grades [n(%)]					χ^2^ = 156.3	< 0.001
High adherence (≥75%)	105 (30.9)	169 (49.7)	218 (64.1)	248 (72.9)		
Moderate adherence (50–74%)	183 (53.8)	143 (42.1)	110 (32.4)	84 (24.7)		
Low adherence (<50%)	52 (15.3)	28 (8.2)	12 (3.5)	8 (2.4)		

Symptom total score, compliance rate: Repeated measures ANOVA (Greenhouse–Geisser correction, ε = 0.89); Compliance rate = (actual score on the scale ÷ 60) × 100%.

### 3.3 Conditional verification

This study gathered data on symptom burden and exercise compliance using self-assessment questionnaires from patients, which carried the risk of common method bias. Confirmatory Factor Analysis (CFA) was employed to examine the data at each time point (T1-T4). The method involved forcibly combining the 8 items of the symptom burden scale and the 15 items of the exercise compliance scale at each time point into a single factor model, and the severity of common method bias was assessed through model fit indices. The analysis results are presented in Table 3. The single factor model fit indices at each time point did not meet the ideal standard (RMSEA > 0.08, CFI < 0.90, TLI < 0.90). These results suggest that there is no serious common method bias in this study, and the data quality is sufficient for conducting cross-lagged analysis.

**Table 3:** Confirmatory factor analysis results for common method bias (*N* = 340).

Point-in-time	RMSEA	CFI	TLI
T1	0.084	0.815	0.798
T2	0.088	0.843	0.831
T3	0.086	0.852	0.840
T4	0.083	0.858	0.847

RMSEA, Root Mean Square Error of Approximation; CFI, Comparative Fit Index; TLI, Tucker-Lewis Index.

### 3.4 Correlation analysis of symptom burden and exercise adherence of patients at four time points

**Figure 2:**
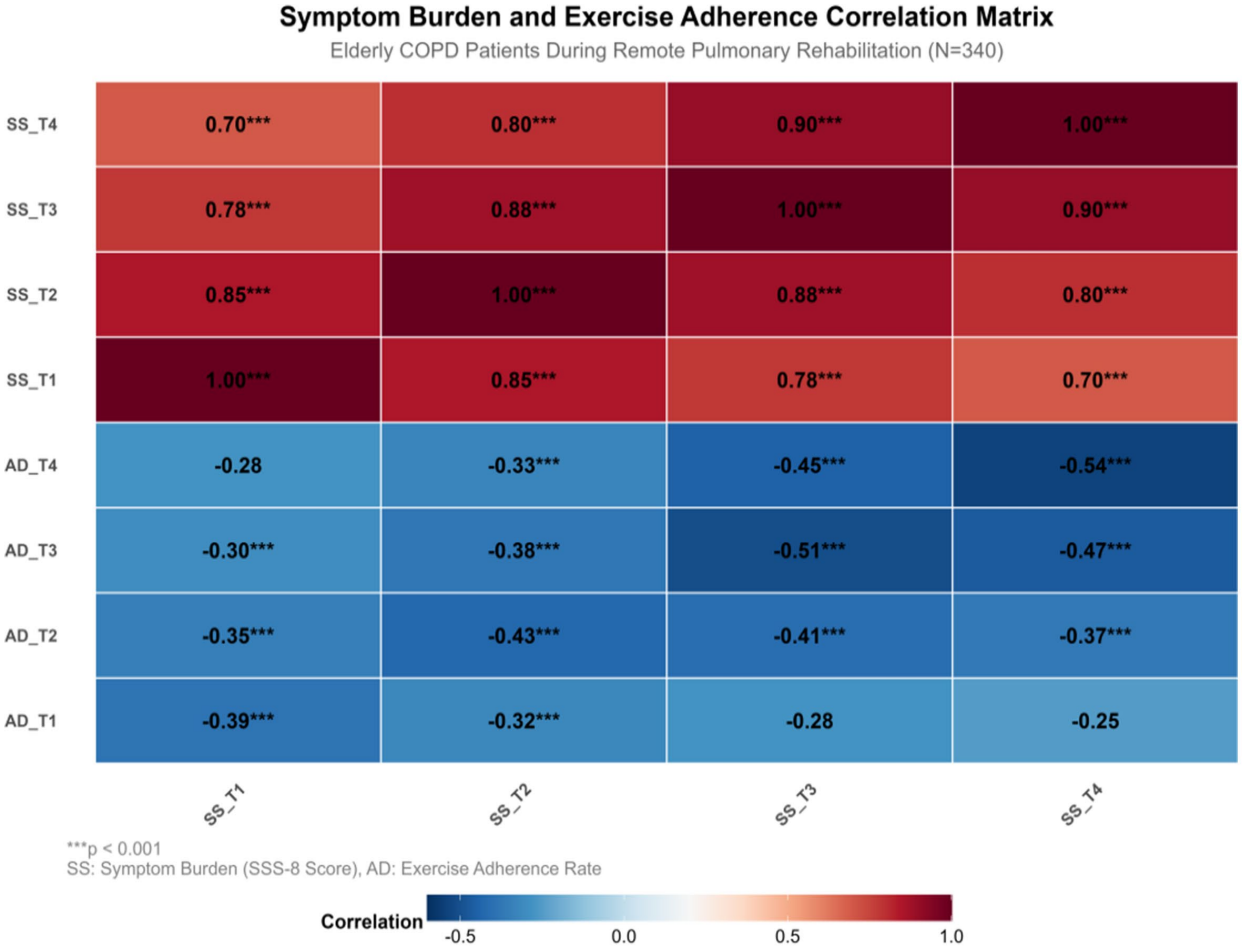
Graph of correlation analysis between symptom burden and exercise adherence at four time points. SS, Symptom Burden(SSS-8 Score); AD, Exercise Adherence Rate. ^***^p < 0.001, ^**^p < 0.01, ^*^p < 0.05. Solid lines represent significant paths; Dashed lines indicate insignificant paths; Red is SS → AD path; The AD→SS path is in blue. Black indicates autoregressive path; Double arrow curves indicate contemporaneous correlations.

Patients with T1, T2, T3, and T4 conditions exhibit a correlation coefficient of −0.39 and −0.43, respectively, for symptom burden and exercise adherence, with values of −0.51 and −0.54 (*p* < 0.001). The correlation for full-time cheng is −0.47, indicating a significant negative correlation between symptom burden and exercise adherence. This negative correlation intensifies over time. Meeting the criteria for cross-lag analysis, the causal time sequence relationship between these variables can be further investigated. For more details, refer to Table 4 and Figure 2.

**Table 4:** Time-point correlation analysis of COPD symptom burden and exercise adherence (*N* = 340).

Point-in-time	Correlation coefficien(r)	95%Cl	*p*	Effect size
T1	−0.39	−0.46–−0.32	< 0.001	Moderate effect
T2	−0.43	−0.50–−0.36	< 0.001	Moderate effect
T3	−0.51	−0.57–−0.45	< 0.001	Large effect
T4	−0.54	−0.60–−0.48	< 0.001	Large effect
Full-time	−0.47	−0.51–−0.43	< 0.001	Large effect

Effect size criteria: |r| ≥ 0.1 was considered as a small effect. |r| ≥ 0.3 was considered moderate effect. Large effects were defined as |r| ≥ 0.5.

### 3.5 Cross-lagged analysis of the relationship between symptom burden and exercise adherence in patients at four time points

**Figure 3:**
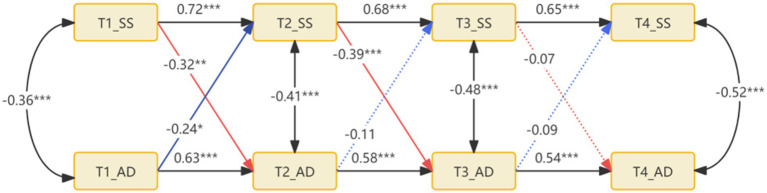
Standardized coefficient diagram of cross-lagged model of patients’ symptom burden and exercise adherence.

Age, sex, GOLD grade, and baseline 6MWD grouping were included as control variables in this study. The model’s fitting indexes were as follows: χ^2^/df = 1.48, RMSEA = 0.089, CFI = 0.978, TLI = 0.970, SRMR = 0.029. The results of the cross-lag model indicated that symptom burden had a predictive effect on exercise adherence: at the early stage (T1-T2), the baseline symptom burden significantly and negatively predicted 5-week exercise adherence (*β* = −0.32^**^); at the middle stage (T2-T3), the 5-week symptom burden had a stronger predictive effect on 9-week compliance (*β* = −0.39^***^). However, the predictive effect of the 9-week symptom burden on 12-week compliance was not significant (*β* = −0.07, *p* > 0.05). Conversely, exercise compliance in the early stage (T1-T2) significantly reduced the subsequent symptom burden (*β* = −0.24^*^), whereas the prediction effect in the middle and late stages (T2-T4) was not statistically significant (|β| ≤ 0.11, *p* > 0.05). Show in Figure 3.

## 4 Discussion

Longitudinal modeling revealed that the symptom burden of older COPD patients during tele-pulmonary rehabilitation exhibited a three-stage dynamic trajectory characterized by “rapid remission, plateau fluctuation, and steady state maintenance.” The total symptom burden during the early rapid remission period (0–5 weeks) decreased significantly from 13.5 ± 3.8 at baseline to 11.2 ± 3.5. The proportion of patients with severe symptoms decreased by 62.3%, from 13.8 to 5.0%. There are two possible reasons for this. On the one hand, remote pulmonary rehabilitation includes regular online symptom interrogation by medical staff, which helps to reduce the risk of acute hypoxia and interrupt the vicious cycle of dyspnea and fear (24). On the other hand, video-guided respiratory retraining activates the prefronto-limbic system pathway, enhancing exercise tolerance and alleviating patients’ fear of exercise (25). Regular exercise enhances diaphragmatic strength and decreases hyperinflation in patients with COPD, establishing the physiological foundation for a reduction in symptom burden (26, 27). During the medium-term plateau fluctuation period (5–9 weeks), the symptom score remained stagnant at 9.8 ± 3.1, and the proportion of patients experiencing highly symptomatic episodes rebounded by 16.5%. It can be considered that the patients experienced fatigue, which triggered a vicious cycle of fatigue and dyspnea (28). During the later homeostatic maintenance period (9–12 weeks), the symptom burden remained stable at 8.4 ± 2.9, and 78.5% of the patients experienced mild to moderate symptoms, potentially due to the behavioral internalization effect. Exercise compliance sustained for over 9 weeks enhanced self-regulation ability, significantly increasing the threshold of symptom tolerance (29), While deepening one’s understanding of the disease, such as identifying warning signs and symptoms, helps patients intervene earlier during mild discomfort and avoid the cumulative aggravation of symptoms.

This study revealed that the exercise compliance of olderly COPD patients during remote pulmonary rehabilitation exhibited a three-stage evolution trajectory characterized by “rapid increase, platform fluctuation, and high maintenance.” This was specifically manifested as an early rapid improvement period (0–5 weeks), during which the proportion of patients with high compliance increased from 30.9 to 49.7%. The average compliance rate increased from 70.3 ± 14.7% to 75.7 ± 13.2% (*p* < 0.001). Although the initial symptom burden of patients was heavy at this stage, low-intensity exercises such as breathing training and slow walking in remote interventions brought slight improvements, including a reduction in the frequency of shortness of breath and an improvement in daily activity tolerance. This immediate experience of “alleviation of discomfort after exercise” helped to break the fear of “exercise aggravating symptoms,” initially establishing the perception that “exercise is beneficial,” and promoting the compliance to increase from baseline. In the middle stage (5–9 weeks). The high compliance rate decreased to 29.0%, while the proportion of patients with low and medium compliance increased to 32.4%. There are two bottlenecks at this stage. On the one hand, there is a physiological bottleneck: mitochondrial dysfunction leads to an increase in blood lactic acid after exercise, resulting in the accumulation of fatigue and interruption of exercise (30). On the other hand, there may be a technical bottleneck. Some patients have insufficient digital literacy and experience difficulty operating smart devices, which leads to an increase in the missing report rate of data and weakens the effectiveness of remote supervision (31, 32). During the later maintenance period (9–12 weeks), the compliance rate continued to rise to 72.9%, indicating that exercise had gradually become a daily habit. Patients’ belief in “exercise to maintain health” became more stable, and they could still maintain high compliance even if the improvement of symptoms slowed down.

Based on the cross-lag model, this study found that symptom burden and exercise adherence in the early stage (T1-T2) were mutually causal. It is suggested that the symptom burden of COPD patients at baseline is the antecedent variable of exercise compliance, and the moderate to severe level of symptom burden (such as dyspnea, fatigue, joint pain, etc.) is the core physiological barrier that prevents patients from participating in exercise (33, 34). Its phasic decline directly creates the basic conditions for the improvement of exercise compliance. Firstly, with the advancement of remote rehabilitation interventions, the gradual improvement of lung function, such as the reduction of airway resistance and the improvement of ventilation efficiency, significantly reduces the frequency and severity of patients’ dyspnea during exercise (35). This experience of “exercise no longer being accompanied by strong discomfort” will directly reduce patients’ fear of exercise, making them more willing to adhere to the rehabilitation plan and complete their training. This is manifested as increased compliance. The improvement in exercise compliance can alleviate the symptom burden of patients (*β* = −0.24). Following exercise, a vastus lateralis muscle biopsy reveals that PGC-1α expression is increased, enhancing oxidative phosphorylation (36), Lactate clearance is accelerated, and respiratory muscle fatigue is delayed (37), and the maximum oxygen uptake of patients is gradually increased, which makes it easier to meet the oxygen demand in daily activities. Thus, the onset of dyspnea can be reduced, and a positive cycle of “exercise persistence → improvement of respiratory function → alleviation of symptoms” can be formed. During the middle stage (T2-T3), symptom burden negatively predicted exercise compliance (β = −0.39), with a decrease in symptom burden correlating with an increase in patient exercise compliance. The analysis indicated that the alleviation of dyspnea and exercise fear, coupled with the patient’s willingness to engage in exercise, were the primary reasons for the improvement in exercise compliance. The relief of symptom burden corresponded with an increase in the 6-min walk distance. Successful movement experiences enhance dopamine release in the nucleus accumbens, reinforcing positive behavioral feedback (38, 39). This study found that symptom burden and loss of exercise adherence could predict each other in the later stage of rehabilitation (T3-T4) (path coefficient | *β* | < 0.10, *p* > 0.20). This phenomenon can be attributed to the stabilization of the patient’s physiological functions and symptom status, resulting in a weakened dynamic relationship between them due to “diminishing marginal benefit.” On one hand, the symptom burden had been reduced to a relatively stable level. Following the initial 9 weeks of intervention, the core symptoms (dyspnea, fatigue, etc.) of patients had diminished from moderate to severe at baseline to mild or subclinical levels, and the potential for further improvement was limited. At this juncture, minor fluctuations in symptom burden no longer substantially impeded exercise capacity—patients had already developed the physiological reserve to adapt to mild symptoms, such as respiratory muscle endurance and exercise tolerance reaching a plateau, making it challenging for symptom changes to significantly impact exercise compliance. On the other hand, the physiological benefits of exercise have reached a plateau. After the T3 stage, the improvement effect of regular exercise on respiratory function gradually slows down, and no longer produces significant improvement in the early (T1-T2) and middle (T2-T3) stages. When exercise fails to bring more obvious feedback of “symptom relief” or “function improvement,” patients’ exercise motivation is no longer driven by symptom changes, but rather transformed into habitual behavior, so the fluctuation of exercise compliance no longer significantly affects the symptoms.

Based on the dynamic trajectory characteristics of symptom burden and exercise adherence, this study proposes a “three-phase precision intervention” strategy. A systematic review indicates that digital interventions can effectively reduce the risk of acute exacerbation-related hospitalization and readmission in COPD patients, providing a theoretical foundation for the staged intervention strategy proposed in this study (40). In the early stage (0–5 weeks), a two-way collaborative intervention was employed. On one hand, VR natural scene exposure therapy was utilized to alleviate exercise fear (41), and video-guided abdominal breathing training was provided to patients to reduce the risk of acute hypoxia during exercise; on the other hand, family members signed an exercise incentive contract to log rewards daily to enhance the initiation intention. The middle stage (5–9 weeks) is the symptom dominant overcoming period, during which graded resistance training is used to reduce the patient’s lactic acid accumulation and prevent fatigue (42). For patients with low digital literacy, a paper graphic manual combined with remote assistance from their children can be considered to ensure technical accessibility (43). In the later stage (9–12 weeks), behavior homeostasis is solidified, and the mutual aid group can be considered to strengthen the social belonging of patients (44).

This study has several limitations that also indicate directions for future research. The sample representativeness may be limited as all participants were recruited from a single medical center in Nantong using convenience sampling. While this approach ensured feasibility, it may affect the generalizability and external validity of the findings. Future studies should therefore employ multi-center, large-sample randomized sampling for verification. Regarding measurement bias risk, the core variables (symptom burden and exercise adherence) were primarily assessed through self-administered questionnaires. Although practical, this method may introduce recall bias and social desirability bias. To enhance data objectivity and accuracy, future research should integrate multiple objective measurement methods, such as using wearable sensors (e.g., accelerometers) for automatic and continuous monitoring of daily activity levels, or utilizing backend logs from telemedicine platforms to precisely record exercise session completion. Concerning follow-up duration and unmeasured confounding factors, the 12-week follow-up period, while sufficient to capture key changes during early rehabilitation, is inadequate to reveal long-term adherence patterns, seasonal impacts on symptoms, or natural disease progression including relapse patterns in COPD patients. Additionally, important confounding factors such as environmental exposure, nutritional status, and social support were not included, potentially leading to an underestimation of effect sizes. Hence, extending the follow-up period and integrating environmental monitoring data with wearable device data to develop more comprehensive predictive models represent crucial directions for future in-depth exploration of the long-term effects of tele-rehabilitation.

## 5 Conclusion

This study revealed, through longitudinal tracking, that olderly patients with COPD experienced a moderately severe level of symptom burden during remote pulmonary rehabilitation. Exercise adherence was at a medium level. Both symptom burden and exercise adherence exhibited a rising trend during the 12-week recovery period, characterized by a three-phase dynamic trajectory. However, a downward trend was also observed. The interaction between symptom burden and exercise compliance was time-dependent. In the early stage of rehabilitation, symptom burden was the primary predictor of exercise compliance, while the reverse prediction was not significant. Clinical medical staff should carry out precise interventions according to the characteristics of the three stages when implementing remote pulmonary rehabilitation.

## Data Availability

The original contributions presented in the study are included in the article/supplementary material, further inquiries can be directed to the corresponding author.
